# Supplementary material for: The shaping of social and symbolic capital during the transition to farming in the Western Mediterranean: Archaeological network analyses of pottery decorations and personal ornaments
Source: PLoS One. 2023 Nov 8;18(11):e0294111. doi: 10.1371/journal.pone.0294111 (PMC10631656; doi:10.1371/journal.pone.0294111)
Supplement: S1 File — Supplementary information for cultural diversity calculation; descriptive statistics definition and application; extra supporting results. (PDF) [file pone.0294111.s003.pdf]

The shaping of social and symbolic capital during the transition to farming in the Western Mediterranean: archaeological network analyses of pottery decorations and personal ornaments

Daniel Pereira[1*], Claire Manen[2], Solange Rigaud[1]

[1] CNRS, Université Bordeaux, Ministère de la Culture, PACEA, UMR 5199, Pessac, France

[2] CNRS UMR5608 TRACES. Université de Toulouse Jean-Jaurès, Maison de la Recherche, Toulouse, France

S1 Supplementary Information. Method and results.

Contents

Methods 1

Supplementary Text A: Cultural diversity 1

Results 5

Supplementary Tables 5

Supplementary Figures 6

Supporting Information 7

Methods

Supplementary Text A: Cultural diversity

Our sample is constituted of two previously published datasets [1]: pottery decorative techniques count data and bead-type association presence/absence data.

The Brainerd-Robinson (BR) coefficient of similarity [2–4] is commonly used in archaeology [5–7] and more specifically for pairwise comparisons of count data. In this study, we use a rescaled version of the BR [4,8,9], outputted in the interval [0, 1] (worksheet A in S2 Supplementary Information), to build the pottery decorative techniques similarity matrix. The Jaccard similarity index [10,11] is another commonly used index in archaeology [1,12] , more specifically for the pairwise comparison of presence/absence data. This index outputs results in the interval [0, 1] (worksheet G in S2 Supplementary Information) [4,10,11,13] and was used to build the personal ornaments similarity matrix.

Supplementary Table 1 - Descriptive statistics definitions and applications

	Statistic	Definition	Application
**Node metrics** [4,8,9,14–16]	(weighted) Degree centrality	Defined as the total weight of all connections for one node (minus 1 to remove self-loops).	Identifies the nodes with higher total edge weight.
	(weighted) Eigenvector centrality	Defined as a node's weighted degree centrality divided by the weighted degree centrality of all the nodes to which it connects.	Is a measure of a node's "social" importance in a network.
	(weighted) Betweenness centrality	Defined as the fraction of all shortest paths between other pairs of nodes that pass through that node, weighted by the strength of the edges on those paths, which in weighted networks equals the higher the edge weights the "shorter" it is.	Identifies nodes that bridge less connected parts of the network.
**Graph metrics** [4,9,14–17]	Centralization metrics	Centralization is calculated by taking the sum of the difference in centrality between the node with the highest centrality and every other node, dividing by the maximum possible in centrality. Calculated for degree, eigenvector and betweenness weighted centralities, all normalized to graph size.	Measure of preponderance of the network to be centralized in one or few nodes. Range [0, 1].
	Network density	Defined as the ratio of existing edges in a network over the maximum number of possible edges for the network.	Describes how densely connected a network is. Range [0, 1].
	Cluster coefficient (global average transitivity)	Defined as the number of closed triplets over the total number of triplets in a network.	A measure of the tendency for nodes to connect to each other. Range [0, 1].

	Network interval statistics	Cultural similarity indices, geographical and chronological distances minimum, mean and maximum values calculated for the threshold matrices.	Describes the intervals in which all the plotted edges fall within the network.
	Similarity radius	Calculated as the average ratio of geographical distance over cultural similarity for each archaeological culture.	Indicates the geographic distance radius in which the cultural similarity is maintained between pairwise occupations within an archaeological culture.
	Mantel and Partial Mantel tests [18]	Defined as the correlation between various distance matrices.	Indicates the correlation level between two distance matrices. Range [0, 1].

All the statistics presented above were calculated in the *R* platform [19] using an adapted and extended version of the code presented by Brughmans and Peeples [8,9,20]. All the statistics were performed, except when indicated otherwise, using the threshold matrices. In the main body, for brevity, we refer to the centralities without making reference to them being weighted, even though all calculations were made as such. The code can be found in S1 Code.

Results

Supplementary Tables

Supplementary Table 2 - Pottery decorative techniques and personal ornaments full and time sequence ASN's summary statistics

Test	Pottery decorative techniques			Personal ornaments		
	Full	TS 1-2	TS 2-3	Full	TS 12	TS 23
**Nodes**	44	37	32	46	37	37
**Edges**	231	183	96	217	117	151
**Cluster coef.**	0.716852	0.730209	0.783005	0.546955	0.513402	0.620690
**Net. density**	0.244186	0.274775	0.193548	0.209662	0.175676	0.226727

Supplementary Table 3 - Pottery decorative techniques and personal ornaments ASN's network interval statistics

	Pottery decorative techniques			Personal ornaments		
	Max	Mean	Min	Max	Mean	Min
**Cult sim**	0.944639	0.609089	0.441387	1	0.260285	0.161290
**Geo dist**	1424.763310	347.592284	0	1540.752	262.361042	0
**Chrono dist**	775	211.082251	0	950	309.976959	0

Supplementary Table 4 - Pottery decorative techniques and personal ornaments ASN's intra-cultural summary statistics.

Culture Complex	Pottery decor. tech.		Pers. Ornaments	
	Net density	Cluster Coef.	Net density	Cluster Coef.
IMP	0.196970	0.666667	0.4	0.6
CARD	0.733333	0.89375	0.2	0.468208
EPIC	0.609524	0.795113	0.191176	0.5

Supplementary Table 5 - Pottery decorative techniques and personal ornaments ASN's intra-cultural network interval statistics. Calculate for threshold matrices.

	Pottery decorative techniques								
	Cultural Sim.			Geographical dist.			Chrono dist.		
	Max	Mean	Min	Max	Mean	Min	Max	Mean	Min
**IMP**	0.944639	0.124297	0	1266.8079	419.7815	0	125	76.9231	0
**RPC**	0.828272	0.550799	0	208.2986	127.6795	10.0626	200	125	50
**LCC**	0.908003	0.400436	0	226.0357	85.2066	3.4571	440	140.2174	0
**VC**	0.743915	0.743915	0.743915	42.8878	42.8878	42.8878	50	50	50
**LCE**	0.902253	0.381030	0	313.0439	133.8864	0	525	187.7660	0
**VE**	0.550987	0.550987	0.550987	42.8878	42.8878	42.8878	75	75	75
	**Personal ornaments**								
	**Cultural Sim.**			**Geographical dist.**			**Chrono dist.**		
	**Max**	**Mean**	**Min**	**Max**	**Mean**	**Min**	**Max**	**Mean**	**Min**
**IMP**	0.428571	0.235931	0.166667	642.7320	390.2074	0	150	87.5	0
**TyC**	0.666667	0.338333	0.2	0	0	0	0	0	0
**RPC**	0.25	0.222222	0.166667	171.3141	69.1913	11.0002	175	100	0
**LCC**	0.5	0.274074	0.166667	194.4059	72.5952	0	850	333.3333	0
**VC**	0.275862	0.275862	0.275862	42.8709	42.8709	42.8709	50	50	50
**LCE**	0.714286	0.269231	0.166667	239.7789	98.4818	0	525	200	0
**VE**	-Inf	NA	Inf	-Inf	NA	Inf	-Inf	NA	Inf

Supplementary Table 6 - Pottery decorative techniques and personal ornaments time sequence datasets ASN's cultural similarity, geographical and chronological distances plotted edges intervals.

		Pottery decorative techniques			Personal ornaments		
	**Period**	**Max**	**Mean**	**Min**	**Max**	**Mean**	**Min**
**Cult sim**	***TS 1-2***	0.944639	0.606790	0.441387	0.666667	0.2830195	0.190476
	TS 2-3	0.908003	0.703713	0.580537	1	0.269864	0.161290
**Geo dist**	***TS 1-2***	1424.763310	384.732808	0	1400.987404	293.660803	0
	TS 2-3	811.418150	281.860727	0	762.9186	146.065288	0
**Chrono dist**	***TS 1-2***	775	210.9836	0	950	260.3846154	0
	TS 2-3	500	170.1562	0	850	279.668874	0

Supplementary Figures

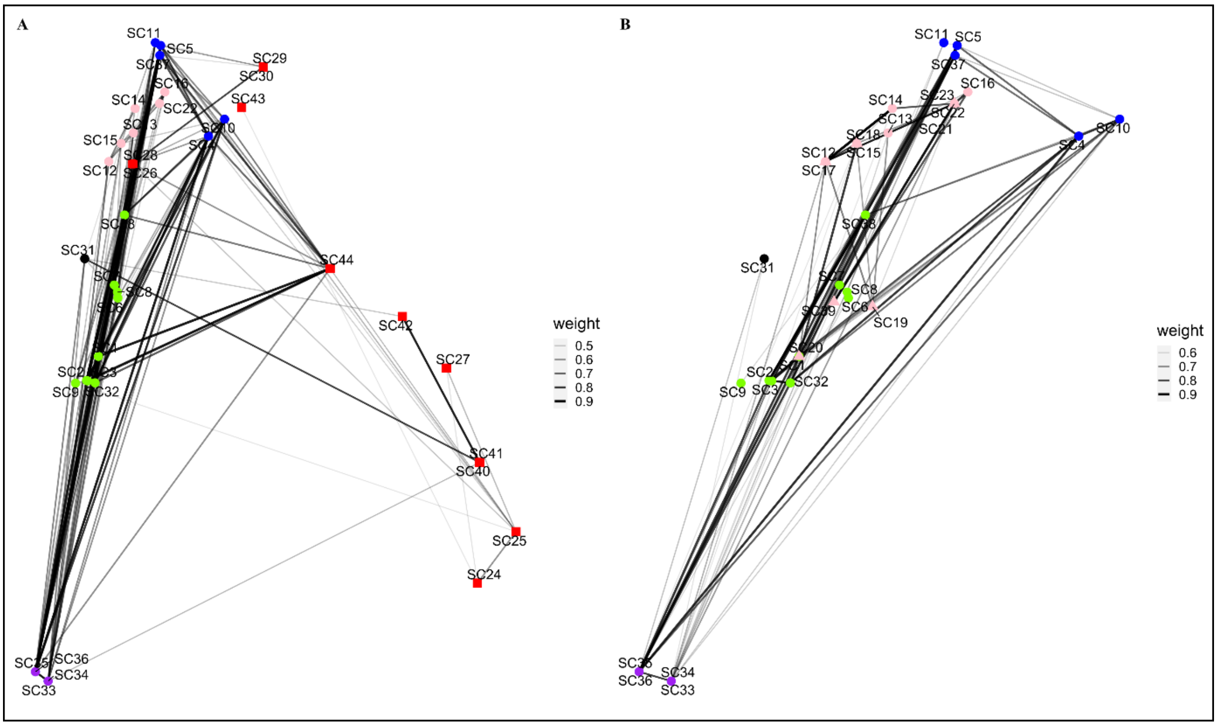

Supplementary Figure 1 - Archaeological Similarity Network (ASN) for pottery decorative techniques time sequence analysis, plotted using each occupation's geographical coordinates. a) time sequence 1-2 network. b) time sequence 2-3 network. ASN's edge weight (thickness) caption to the right of corresponding network. Marker colours: red – IMP; blue – RPC; green – LCC; cyan – VC; pink – LCE; purple – VE; black – La Balma Margineda. Marker shapes: square – 1st period; round – 2nd period; triangle – 3rd period. Due to geographical coordinates overlap, not all archaeological location markers are visible.

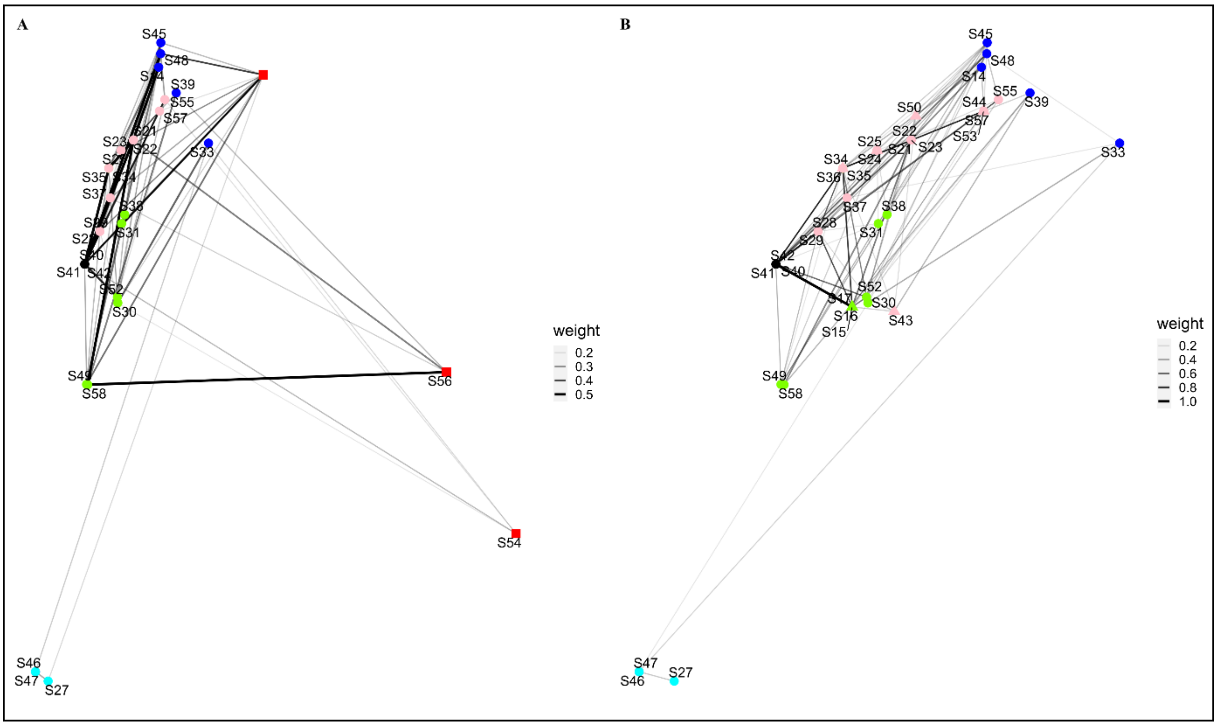

Supplementary Figure 2 - Archaeological Similarity Network (ASN) for personal ornaments time sequence analysis, plotted using each occupation's geographical coordinates. a) time sequence 1-2 network. b) time sequence 2-3 network. ASN's edge weight (thickness) caption at the right of corresponding network. Marker colours: red – IMP; golden – TyC; blue – RPC; green – LCC; cyan – VC; pink – LCE; purple – VE; black – La Balma Margineda. Marker shapes: square – 1st period; round – 2nd period; triangle – 3rd period. Due to geographical coordinates overlap, not all archaeological location markers are visible.

Supporting Information

S1 Dataset. Archaeological site dataset. Database of the archaeological sites, layers, variables, and radiocarbon dates used in the analysis.

S1 Supplementary Information. **Method and results.** Supplementary information for cultural diversity calculation; descriptive statistics definition and application; extra supporting results.

S2 Supplementary Information. Results. Cultural similarity, geographical and chronological distance matrices; complete node centralities score tables.

S1 Code. Analyses code protocol.

References

1. Rigaud S, Manen C, García-Martínez de Lagrán I. Symbols in motion: Flexible cultural boundaries and the fast spread of the Neolithic in the western Mediterranean. PLOS ONE. 2018;13: e0196488. doi:10.1371/journal.pone.0196488

2. Brainerd GW. The Place of Chronological Ordering in Archaeological Analysis. American Antiquity. 1951;16: 301–313. doi:10.2307/276979

3. Robinson WS. A Method for Chronologically Ordering Archaeological Deposits. American Antiquity. 1951;16: 293–301. doi:10.2307/276978

4. Brughmans T, Peeples MA. Network Science in Archaeology. Cambridge: Cambridge University Press; 2023. doi:10.1017/9781009170659

5. Birch J, Hart JP. Conflict, Population Movement, and Microscale Social Networks in Northern Iroquoian Archaeology. American Antiquity. 2021;86: 350–367. doi:10.1017/aaq.2021.5

6. Hart JP, Shafie T, Birch J, Dermarkar S, Williamson RF. Nation Building and Social Signaling in Southern Ontario: A.D. 1350–1650. PLOS ONE. 2016;11: e0156178. doi:10.1371/journal.pone.0156178

7. Hart JP, Engelbrecht W. Northern Iroquoian Ethnic Evolution: A Social Network Analysis. Journal of Archaeological Method and Theory. 2012;19: 322–349. doi:10.1007/s10816-011-9116-1

8. Peeples MA. A Brief Introduction to Archaeological Networks in R. 2019. Available: http://www.mattpeeples.net/netintro.html

9. Brughmans T, Peeples MA. Online Companion to Network Science in Archaeology. 2023. Available: https://archnetworks.net/

10. Jaccard P. Étude comparative de la distribuition florale dans une portion des Alpes et des Jura. Bull Soc Vaudoise Sci Nat. 1901;37: 547–579.

11. Jaccard P. The Distribution of the Flora in the Alpine Zone. The New Phytologist. 1912;11: 37–50.

12. Gibbon E, Knobloch P, Jennings J. Complicating an early state: a social network analysis of agents in Wari art (*c.* AD 700–850). Antiquity. 2022;96: 646–661. doi:10.15184/aqy.2022.53

13. Bernabeu Aubán J, Lozano S, Pardo-Gordó S. Iberian Neolithic Networks: The Rise and Fall of the Cardial World. Frontiers in Digital Humanities. 2017;4: 7. doi:10.3389/fdigh.2017.00007

14. Freeman LC. Centrality in social networks conceptual clarification. Social Networks. 1978;1: 215–239. doi:10.1016/0378-8733(78)90021-7

15. Golbeck J. Chapter 3 - Network Structure and Measures. In: Golbeck J, editor. Analyzing the Social Web. Boston: Morgan Kaufmann; 2013. pp. 25–44. doi:10.1016/B978-0-12-405531-5.00003-1

16. Iacobucci D, McBride R, Popovich DL. Eigenvector Centrality: Illustrations Supporting the Utility of Extracting More Than One Eigenvector to Obtain Additional Insights into Networks and

Interdependent Structures. Journal of Social Structure. 2017;18: 1–23. doi:doi:10.21307/joss-2018-003

17. Hansen DL, Shneiderman B, Smith MA, Himelboim I. Chapter 3 - Social network analysis: Measuring, mapping, and modeling collections of connections. In: Hansen DL, Shneiderman B, Smith MA, Himelboim I, editors. Analyzing Social Media Networks with NodeXL (Second Edition). Morgan Kaufmann; 2020. pp. 31–51. doi:10.1016/B978-0-12-817756-3.00003-0

18. Mantel N. The Detection of Disease Clustering and a Generalized Regression Approach. Cancer Research. 1967;27: 209–220.

19. Team RC. R: A Language and Environment for Statistical Computing. Vienna, Austria: R Foundation for Statistical Computing; 2022. Available: http://www.R-project.org/

20. Csardi, Gabor, Nepusz, Tamas. The igraph software package for complex network research. InterJournal. 2006;Complex Systems: 1695.
